# Expert Medical Testimony1Presidential address delivered at the thirty-ninth annual meeting of the Medical Association of Central New York, held at Syracuse, October 16, 1906.

**Published:** 1907-01

**Authors:** David M. Totman

**Affiliations:** Syracuse. N. Y.


### Expert Medical Testimony.[1]

By DAVID M. TOTMAN, M. D., Syracuse. N. Y.

PERMIT me to express to you my appreciation of the honor you have conferred upon me, in making me your presiding officer of this the thirty-ninth annual meeting of this association. In reviewing the names of the men who have been presidents my attention has been called to the number of honorable names in this list; men who have added to the working resources of the medical profession, men who were great teachers and exemplars of all that is best in the medical profession.

I had planned, after my election, to write a paper on "Flat-foot"—a subject on which I have worked for several years, and Dr. Weigel who was present and read a most excellent paper at the Buffalo meeting, promised to follow me with a paper, giving his researches in this very field, and in which he laid the beginnings of the fatal disease which ended his life. Truly he laid down his life for others. I wish to call your attention to a subject which we will have to meet soon—namely, the future status of the association. Are the meetings of the district branches of the state society to affect our meetings?

In accordance with time-honored custom wisely established, it becomes one of the functions of the president to present an address. With a whole year before him he should and is expected to present something of his best endeavor in some line of work which lies close to the heart of the profession. I therefore beg your indulgence for a few minutes while I present the subject of

EXPERT MEDICAL TESTIMONY.

It is necessary for me to outline briefly the history of a negligence suit tried in the supreme court in the city of Syracuse which culminated in a jury verdict for the plaintiff of $20,000 which sum was reduced by order of the trial judge to $15,000, and which is now in the appellate court, but has not yet been argued.

The plaintiff in the case was a young unmarried woman of Irish descent, whose father showed evidences of seriously impaired health, and it was asserted that the mother had suffered serious mental and nervous disease, while the young woman herself was plainly of a highly nervous temperament. One evening

---

1. Presidential address delivered at the thirty-ninth annual meeting of the Medical Association of Central New York, held at Syracuse, October 16, 1906.

she fell in a subway trench, which was two or three feet deep, being under construction for a telephone company. The accident happened late in September, 1905, about 8:30 P. M. The usual curious crowd collected. Dr. Alfred Mercer was summoned and examined carefully the patient while she was sitting nearby the place of accident. His statement to me was that some general excitement prevailed, and that the young woman was in a state of nervous perturbation. She was taken soon afterward in a carriage to her home, and another physician was summoned. It appeared later that there was a cut on or just below left knee, which never left even a scar. The description given of this wound was that it was about an inch long and one-fourth inch deep. Also, that there was a small swelling on the back of the head, which rapidly disappeared.

It appears that on that same evening at ten or eleven o'clock the physician told the patient that the knee injury was a serious one. That she would have a stiff and impaired joint, and he put on a plaster cast, and the same was worn for thirteen days. It was then taken off and again reapplied and worn for some days longer. This physician committed suicide a little time after this, and other physicians were employed, but were not called into court. It appears that within less than three weeks a suit for $25,000, was begun, and the patient was on crutches about the house for some months. In the latter part of February, 1906, another physician was called, and on or about March 8, the woman had some convulsions, which lasted about twenty-four hours, and following this she became unconscious. The case was duly heralded in the newspapers through many months, as the wonderful sleeping girl. The house was thronged by many people prompted by curiosity in quest of the marvelous.

I first saw the patient, being called as a consultant on March 28, and made a second visit on or about April 8 following. I made a careful examination and study of the case on these two visits. Again, on April 22, I saw and critically examined the case as one of two physicians appointed or chosen by the defendant, with the direction and approval of the court. The attending physician was present at this examination. The case was gone over in the minutest detail. In my opinion my visits as a consultant gave me an opportunity of great value, to obtain information regarding the case. I was thus enabled to consider the case solely in the interest of the patient, at a time when the question of her life was in the balance, and when, unless something was done and right promptly, she would die, for I was informed that she had taken no nourishment for three weeks, and was actually suffering from starvation, which her appearance indicated.

I will now read an abstract of the official minutes of the stenographer, as taken in the trial.

Q. Give the evidences first, and then tell us what it is. (Her state).

A. The condition of the patient, the mental condition of the patient, the mental signs, that is, the seeming sleeping condition, the closed eyes, the quivering lids, the following of the lids with the eye ball when the eye was opened, the loss of sensation over one-half of the body, the rigid condition of the left leg, the slightly exaggerated condition of the reflexes in the right leg, and also in the left, the dusky appearance of the skin on the arm from wrist down, the fact that the left leg was involved alone, were all physical evidences of functional disorder.

Q. What kind of disorder?

A. Hysteria.

The Court: Just give the causes of hysteria.

The Witness: The causes of hysteria fundamentally cannot be given; certain manifestations can be given, but as to the fundamental causes which produce hysteria, it reaches further back than the incident which brings it out.

Q. Are there certain classes of persons who are more predisposed to hysteria than others?

A. There are.

Q. What are they?

A. Those of a nervous, highstrung, emotional, excitable temperament; the active factors in producing hysteria, or hysterical manifestations are, excitement, emotion, anxiety, fatigue, worry, fright, and suggestion.

Q. What do you mean by suggestion?

A. Suggestion is the introduction into the mentality—into the mind of an individual,—of certain thoughts, which are more or less fixed, either by the speech of another person or by something seen or something felt, which produces a fixed and permanent, more or less permanent sensation of pain, and more or less functional activity of certain portions of the body.

Q. Upon the question of suggestion?

A. That suggestion, that fixed idea,—the application of a splint to a knee or a joint in a hysteric individual, for example,—will produce a stiffness in that joint every time.

Now I think I have laid sufficient foundation to proceed to the essential part of this paper—namely, the consideration of the question as to what is to be done with expert medical testimony.

First, I wish to make two or three preliminary statements, to show simply what has given the trend to my thoughts. I well remember the effect of Dr. Sefton's paper read in a meeting at Rochester a few years ago, and the full and animated discussion of the subject of expert medical testimony which followed. Within a year in a conversation with one of the most learned and distinguished members of the bar in my acquaintance, he strongly advised me never to go into a medico-legal case again. He said there was neither honor nor glory nor anything else to get out of it, but to get my name smirched and dishonored. I say to you now that the conditions which surround the giving of expert testimony in our courts have become well nigh intolerable, considering the attitude of the public press, the insulting innuendos of graft, and the charge of open perjury committed by medical men on the witness stand; hence I affirm with emphasis that these conditions are not getting better, but have become intolerable.

I had a discussion with my confrère in this case, as to whether we would go into it or not. In seeking some relief for the conditions which burden the profession with disgrace, I offer briefly the following suggestions, which have grown in my mind from a careful study of this case.

First of all, I see no relief in trying to reach a change in the method of a jury trial. Jury trials are to be had, and it will take centuries of education and evolution for a jury to ever comprehend such expert testimony as was given in this trial of which I have given you a little part, so I say, no matter how true or perfect the testimony which can be presented to a jury may be, the verdict will not be influenced one whit.

The statement which I now offer, I make with diffidence and hesitation; it is that some facts which have come to my personal knowledge, convince me that even the learned judges are not always able to appreciate the bearings of expert medical evidence such as was given in this case. I do not mention this in the way of criticism nor in a captious spirit; I am only considering the subject in the line of facts. I hold this, that hysteria is a subject of such a nature that neither juryman nor a judge, however learned he may be, is able to execute justice when such a subject is under consideration. The learned judge can have access to all medical libraries, he may read every treatise that was ever written, yet his knowledge is as nothing. The only man who can appreciate and execute judgment in a case where this disorder of so many different forms of expression is found, is a well educated and trained medical man, with at least fifteen or twenty years of study, observation, and management of hysteria; bedside treatment first, hospital treatment, home treatment, are all essential; therefore, the man to pass upon the value of expert medical testimony must be a man who is able to understand such evidence in its entirety.

My first thought was that a way to meet this matter was through and by proper legislation,—to have one additional member of the Appellate Court, a properly qualified medical man, whose sole duty should be to pass upon medico-legal testimony. Further consideration leads me to go a step farther in my suggestion, and that is to have the power given by legislation to the Appellate Courts in our state, to name a board of three members for each Appellate Division of properly qualified physicians, of at least fifteen or twenty years practice, to serve for a period of fourteen years, whose proper office and duty should be to consider all medico-legal cases, under the jurisdiction of the Appellate Court as advisors to the Court. The judges of each Appellate Court should be authorized to choose the men in their own district, as their acquaintance with the physicians will aid them in the selection of the men best fitted for this work

What will be the results? If there can be a proper review of medico-legal evidence by men competent to sift and analyse the same, no one can tell the ultimate results. Please bear in mind that I have not made mention in a single word of the evidence of the medical experts for the plaintiff. However, in my opinion, a whole volume could be written on it. And I wish it might be published to the medical profession. It would be the most powerful argument which could possibly be advanced in the support of the sum and substance of this paper—namely, that in some way there shall be established a proper review of medico-legal evidence, which I am free to say, does not obtain at the present time.

First, it will be a death blow to ambulance chasers, who have worm-eaten the legal profession quite as badly as any influences have affected the medical profession. Justice will be given in the cases, which is the main function of the case; shrewd lawyers have profited for years from the fact that medico-legal evidence has been measured by bulk and not by quality. The medical profession has a closer and more intimate association with the people than any other profession. I conceive that in the future the medical profession is to have a broader and more useful field, even that it will enter upon the field by shaping and carrying home such legislation, as shall be more clearly under the care and jurisdiction of the most enlightened and progressive profession in the world.

In conclusion I wish to say that I know that this paper has presented the subject matter in but a crude way. One of the greatest teachers I ever had, Professor Eugene Lamb Richards, now emeritus Professor of Mathematics in Yale University, was accustomed at the end of the regular recitation, to throw on the blackboard original propositions in geometry or analytical geometry, and then call for volunteers to solve them, and to him who solved a problem was given a perfect mark. The method of solution was never criticised. You could begin at the back and go forward, or up or down; you could use a pick or shovel; if you got the solution correct, you had the reward,—a perfect mark.

If I have simply stimulated someone else to carry on this work, —if I have blazed even a rude trail,—I shall not have thought and written in vain.

303 MONTGOMERY STREET.

